# The relationship between fasting plasma citrulline concentration and small intestinal function in the critically ill

**DOI:** 10.1186/s13054-014-0725-4

**Published:** 2015-01-20

**Authors:** Alexis Poole, Adam Deane, Matthew Summers, Janice Fletcher, Marianne Chapman

**Affiliations:** Intensive Care Unit, Royal Adelaide Hospital, North Terrace, Adelaide, South Australia 5000 Australia; Discipline of Acute Care Medicine, University of Adelaide, Frome Road, Adelaide, South Australia 5000 Australia; Genetics and Molecular Pathology, SA Pathology, Frome Road, Adelaide, South Australia 5000 Australia

## Abstract

**Introduction:**

In this study, we aimed to evaluate whether fasting plasma citrulline concentration predicts subsequent glucose absorption in critically ill patients.

**Methods:**

In a prospective observational study involving 15 healthy and 20 critically ill subjects, fasting plasma citrulline concentrations were assayed in blood samples immediately prior to the administration of a liquid test meal (1 kcal/ml; containing 3 g of 3-*O*-methylglucose (3-OMG)) that was infused directly into the small intestine. Serum 3-OMG concentrations were measured over the following 4 hours, with the area under the 3-OMG concentration curve (AUC) calculated as an index of glucose absorption.

**Results:**

The groups were well matched in terms of age, sex and body mass index (BMI) (healthy subjects versus patients, mean (range) values: age, 47 (18 to 88) versus 49 (21 to 77) years; sex ratio, 60% versus 80% male; BMI, 25.2 (18.8 to 30.0) versus 25.5 (19.4 to 32.2) kg/m^2^). Compared to the healthy subjects, patients who were critically ill had reduced fasting citrulline concentration (26.5 (13.9 to 43.0) versus 15.2 (5.7 to 28.6) μmol/L; *P* < 0.01) and glucose absorption (3-OMG AUC, 79.7 (28.6 to 117.8) versus 61.0 (4.5 to 97.1) mmol/L/240 min; *P* = 0.05). There was no relationship between fasting citrulline concentration and subsequent glucose absorption (*r* = 0.28; *P* = 0.12).

**Conclusions:**

Whereas both plasma citrulline concentrations and glucose absorption were reduced in critical illness, fasting plasma citrulline concentrations were not predictive of subsequent glucose absorption. These data suggest that fasting citrulline concentration does not appear to be a marker of small intestinal absorptive function in patients who are critically ill.

## Introduction

Nutritional therapy is an integral component of the management of patients admitted to the intensive care unit (ICU) who are critically ill, and the enteral route is preferred to intravenous feeding [1,2]. However, it has recently been demonstrated that enteral nutrient absorption is impaired in a variable, unpredictable and unquantifiable way [3-6]. As adequate energy delivery is important for optimal clinical outcomes, it would be beneficial to be able to identify, in the clinical setting, those patients who have impaired nutrient absorption and be able to quantify this.

Plasma concentration of the amino acid citrulline, which is synthesized mainly in the small intestine, is reduced in specific disease states, including Crohn’s disease, coeliac disease and short bowel syndrome, and this probably reflects reduced functional enterocyte mass [7-14]. However, conflicting results have been reported by researchers who have looked at whether plasma citrulline concentrations predict small intestinal absorptive function in patients with short bowel syndrome [7,9,15]. Plasma citrulline concentrations have been measured in patients who are critically ill, with low concentrations documented [13,14,16], suggesting that critical illness may also be associated with a reduction in functional enterocyte mass [14]. Piton *et al*. reported that very low citrulline concentrations were associated with increased mortality and speculated that these low concentrations may have resulted from gastrointestinal damage [13,14,17]. Although it has been suggested that citrulline concentration may be a marker of small intestinal absorptive function, the relationship between macronutrient absorption and citrulline concentration has not been evaluated in patients who are critically ill. If citrulline concentration were able to accurately identify and quantify impaired small intestinal absorption in patients who are critically ill, such a marker would provide valuable clinical information that would aid in optimising nutritional support. Therefore, the aim of this study was to evaluate the relationship between fasting plasma citrulline levels and small intestinal glucose absorption in critically ill patients and compare the results to healthy subjects.

## Material and methods

Following approval by the Human Research Ethics Committee of the Royal Adelaide Hospital, a prospective observational study involving critically ill patients and healthy subjects was undertaken according to guidelines on research conducted on unconscious patients as outlined by the National Health and Medical Research Council of Australia. Informed written consent for each patient was obtained from the next of kin and from the healthy subjects themselves.

### Participants

Mechanically ventilated (≥72 hours) adult (≥18 years) patients were recruited from the Royal Adelaide Hospital ICU, a 24-bed mixed medical-surgical ICU at a large university-affiliated metropolitan teaching hospital in South Australia. Patients were receiving or were suitable to receive postpyloric enteral feeding. Exclusion criteria included pregnancy, history of diabetes mellitus, previous abdominal surgery, receiving erythromycin at an antimicrobial dose and/or receiving parenteral nutrition.

Healthy adult subjects were recruited from advertisements and from records kept by the Intensive Care Research Unit of persons potentially prepared to volunteer for research studies. Subjects who had previously undergone insertion of a nasogastric tube were preferred, as stress and anxiety relating to tube insertion may affect gastrointestinal function, which would be less likely in individuals with such experience [18]. Healthy subjects were excluded if they were pregnant or breastfeeding, had undergone previous abdominal surgery, had current or previous gastrointestinal disease or were taking medications known to affect gastrointestinal motility. Older, male healthy subjects with a higher BMI were specifically sought in an attempt to match the critically ill population with respect to sex, age and BMI, as it is known that small intestinal function may vary with these parameters [19-21].

### Study protocol

Critically ill subjects were fasted for a minimum of 6 hours prior to study commencement. Those prescribed prokinetic agents had doses held prior to and during the study. A postpyloric feeding tube was inserted using an electromagnetic guidance technique (CORTRAK 2 EAS enteral access system; CORPAK MedSystems, Buffalo Grove, IL, USA), which uses a proprietary feeding catheter that contains a removable stylet with an electromagnetic transmitter, receiver unit and placement monitor [22]. Accurate placement was confirmed based on an abdominal radiograph. An arterial line, part of the routine care of ICU patients, was used for blood-sampling purposes.

The healthy subjects attended the ICU research centre at the Royal Adelaide Hospital at 8:00 am following an overnight fast. After administration of a local anaesthetic throat spray (Co-Phenylcaine Forte Spray; ENT Technologies, Hawthorn East, VIC, Australia), a silicone rubber catheter (Mui Scientific, Mississauga, ON, Canada) that had seven side holes at 1.5-cm intervals and a duodenal feeding port was inserted into the stomach with the aid of tip weights. The healthy subject then assumed the right lateral position to aid migration of the catheter into the duodenum. Accurate placement was confirmed based on antroduodenal transmucosal potential difference. The study commenced once all side holes were confirmed to be in the duodenum [23]. A 20-gauge intravenous cannula was inserted into an antecubital vein to allow blood sampling.

Following confirmation of correct tube placement, all participants received the test ‘meal’. This consisted of 100 ml of liquid nutrient (1 kcal/ml Ensure; Abbott Australasia, Botany, NSW, Australia) mixed with 3 g of 3-*O*-methylglucose (3-OMG) (Sigma-Aldrich, Castle Hill, NSW, Australia). 3-OMG is a synthetic sugar that is absorbed by the same mechanism as glucose but is metabolically inert, meaning that plasma levels of 3-OMG reflect glucose absorption [5]. This meal was infused via the postpyloric tube directly into the small intestine over a period of 6 minutes, using a syringe driver (Alaris GH plus Mk3; CareFusion, Rolle, Switzerland) to provide consistent flow.

### Outcome measures

We recorded descriptive data, including demographic variables, and, for patients, admission diagnosis, disease severity scores (Acute Physiology and Chronic Health Evaluation II score on the day of the study), serum creatinine and 90-day mortality.

Blood samples were drawn at baseline (prior to delivery of the test meal) and at 5, 15, 30, 45, 60, 90, 120, 150, 180, 210 and 240 minutes following the infusion of the test meal. Plasma samples were immediately placed in a cooled storage container and then centrifuged at 3,200 rpm for 15 minutes at 4°C (Universal 320R centrifuge; Hettich, Tuttlingen, Germany). Samples were then decanted and placed in 5-ml collection tubes and stored in a monitored −80°C specimen freezer (MDF-U74V; Sanyo Electric Co, Osaka, Japan) until analysis was performed. Serum samples were kept at room temperature for 30 to 60 minutes to allow clot formation to occur, then centrifuged as above, decanted into 5-ml aliquots and placed into a monitored −80°C specimen freezer. Serum 3-OMG concentrations were measured using liquid chromatography/mass spectroscopy. Citrulline concentrations were determined by stable isotope dilution tandem mass spectrometry (coefficient of variation, <10%). (API 4000; AB SCIEX, Framingham, MA, USA) at the SA Pathology laboratory (Women’s and Children’s Hospital, North Adelaide, SA, Australia) [24].

### Statistical analysis

The area under the 3-OMG concentration curve (AUC) was used to quantify glucose absorption [3] and was calculated using the trapezium rule [25]. Independent samples *t*-tests were used to compare data between the critically ill patients and healthy subjects. Critically ill patients and healthy subjects were considered separately and as a single cohort when analysing for potential relationships between plasma citrulline concentrations and glucose absorption (3-OMG AUC). These relationships were analysed using Pearson’s correlation coefficient to examine a linear association between two normally distributed variables. The two time points selected to measure 3-OMG AUC were 60 minutes postmeal, reflecting early absorption, and 240 minutes postmeal, reflecting overall absorption at the completion of data collection. Analyses were performed using IBM SPSS Statistics version 20.0 software (SPSS, Chicago, IL, USA), and statistical significance was defined as *P* ≤ 0.05.

## Results

Twenty-one critically ill patients and seventeen healthy subjects were recruited into the study. One patient and two healthy subjects had incomplete blood sampling due to line blockage or removal. Their data were excluded, leaving 20 critically ill patients and 15 healthy subjects included in the analyses. The study was well tolerated, with no adverse events. Descriptive data, including demographic variables and illness severity, are presented in Table 1.

**Table 1:** **Demographic characteristics of the patients and healthy subjects** ^**a**^

	**Critically ill patients (** ***n*** **= 20)**	**Healthy subjects (** ***n*** **= 15)**	***P*** **-values**
Age (years)^a^	49 (21 to 77)	47 (18 to 88)	0.80
Sex^b^			0.20
Male	16 (80%)	9 (60%)	
Female	4 (20%)	6 (40%)	
Body mass index^b^ (kg/m^2^)	25.5 (19.4 to 32.2)	25.2 (18.8 to 30.0)	0.84
Admission diagnosis^c^			
Multitrauma	6 (30%)	N/A	N/A
Pneumonia	4 (20%)		
Neurosurgical	3 (15%)		
ARDS	2 (10%)		
Burns	2 (10%)		
Cardiac surgery	1 (5%)		
Pancreatitis	1 (5%)		
Sepsis	1 (5%)		
APACHE II score^d^	18 (4 to 27)	N/A	N/A
Serum creatinine^b^ (μmol//L)	91.2 (31 to 357)	N/A	N/A
90 day mortality^c^	2 (10%)	N/A	N/A

^a^APACHE, Acute Physiology and Chronic Health Evaluation; ARDS, Acute respiratory distress syndrome; N/A, Not applicable. ^b^Mean (range). ^c^Number (percentage). ^d^Median (range).

**Figure 1:**
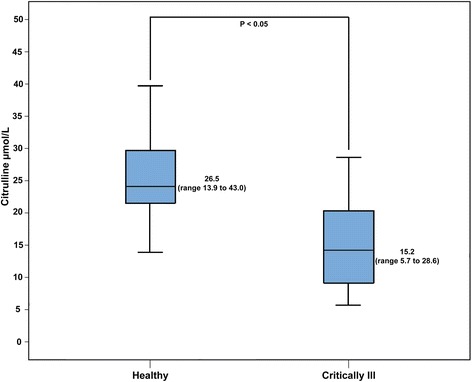
**Fasting plasma citrulline concentrations in healthy subjects and critically ill patients.**

**Figure 2:**
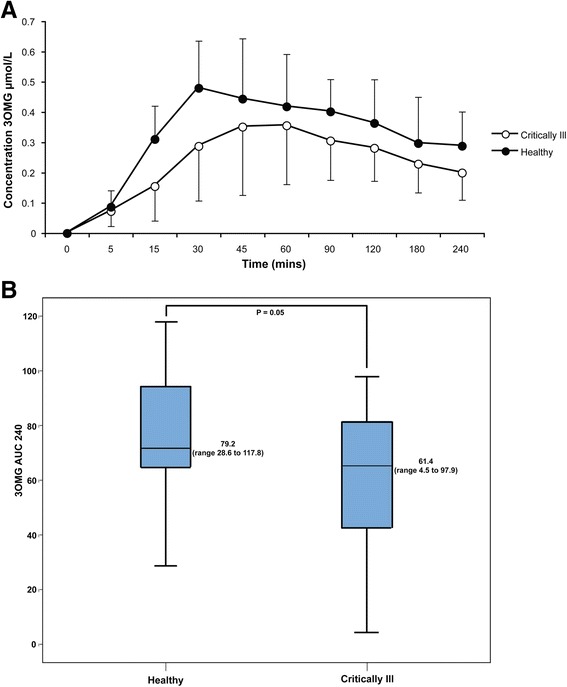
**Comparison of glucose absorption in healthy and critically ill patients. (A)** Glucose absorption assessed using 3-*O*-methylglucose (3-OMG) concentration against time and the area under the 3-OMG curve (3-OMG AUC) in critically ill patients and healthy subjects. **(B)** Total glucose absorption in critically ill and healthy subjects using 3-OMG concentration.

Fasting plasma citrulline concentration was significantly reduced in the critically ill patients compared to the healthy subjects (*P* < 0.01) (Figure 1). Similarly, glucose absorption (3-OMG AUC at 240 minutes postmeal) was reduced in the critically ill patients compared to the healthy subjects (*P* = 0.050) (Figure 2A,B).

**Figure 3:**
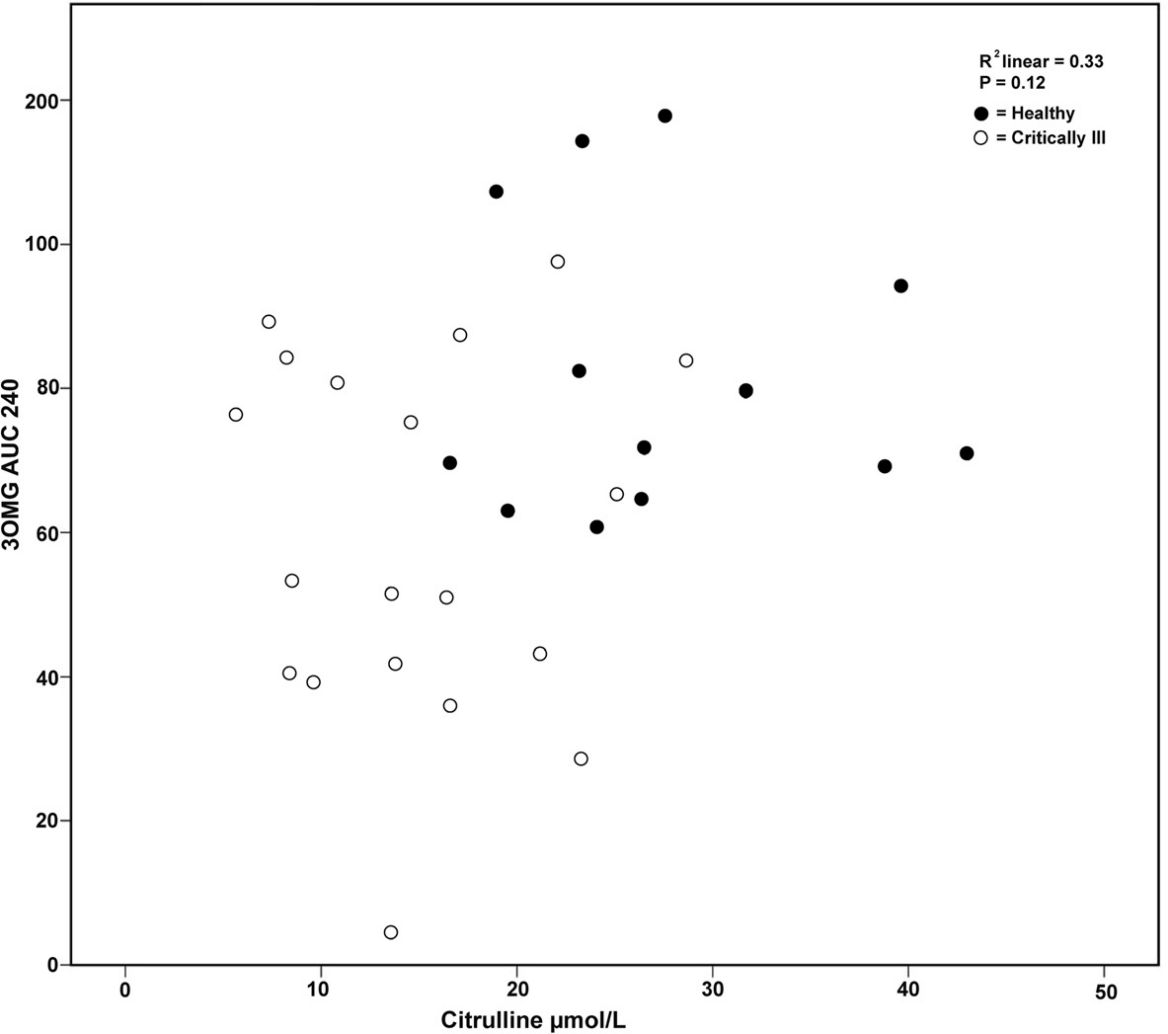
**Scatterplot of glucose absorption and fasting plasma citrulline concentration for the entire cohort.** Data are represented as area under the curve for serum 3**-** *O*-methylglucose concentration (3-OMG AUC) at 240 minutes postmeal.

No association was found between fasting citrulline concentration and subsequent glucose absorption (3-OMG AUC at 60 and 240 minutes postmeal) for the group as a whole (at 60 minutes: *r* = 0.21, *P* = 0.25; at 240 minutes: *r* = 0.28, *P* = 0.12). Similarly, no association was found when the critically ill patients and healthy subjects were considered on their own at either 60 minutes (critically ill patients: *r* = −0.008, *P* = 0.97; healthy subjects: *r* = 0.24, *P* = 0.43) or 240 minutes (critically ill patients: *r* = 0.16, *P* = 0.51; healthy subjects: *r* = −0.04, *P* = 0.90) postmeal. Figure 3 shows a scatterplot of the results for the entire sample at 240 minutes postmeal.

## Discussion

The aim of this study was to examine the relationship between fasting plasma citrulline concentration and small intestinal glucose absorption in critically ill patients and healthy subjects. In so doing, we sought to assess whether citrulline concentration could be used as a biomarker to identify and quantify this aspect of small intestinal function in patients who are critically ill. To our knowledge, this is the first study to examine this relationship. Although citrulline concentrations were reduced and glucose absorption was impaired in the critically ill patients, there was no relationship between fasting citrulline concentration and subsequent glucose absorption. These results suggest that fasting plasma citrulline concentration is not a marker of glucose absorption in critical illness or in health.

The findings related to reduced citrulline concentration and impaired glucose absorption confirm previous findings. Reference values determined in other laboratories for citrulline concentrations are 24 to 50 μmol/L in healthy subjects and 10 to 29 μmol/L in critically ill patients [26], which are consistent with the values reported in our present study [27-36]. Reduced citrulline concentration has been associated with illness severity and with poor outcome [16,17,37]. The data for glucose absorption using 3-OMG concentration are similar to those reported by our group previously [3,4].

Despite both fasting plasma citrulline concentration and overall glucose absorption being reduced in our sample of critically ill patients, we did not find an association between the two. There are a number of possible reasons for this. Although the mechanisms underlying impaired glucose absorption in critical illness are uncertain, they are likely to be multifaceted, with factors including the rate of gastric emptying, intestinal transit time, duodenal and/or jejunal flow events [3,6], the integrity of surface villi and the presence and function of sodium-glucose active transport molecules on intestinal enterocytes [4,38]. In the setting of these multifactorial influences, our results suggest that citrulline concentration may not be sufficiently sensitive to indicate carbohydrate malabsorption or reflect the absorptive capacity of the small intestine. Furthermore, in patients who are critically ill, in whom multisystem dysfunction is commonplace, it is possible that citrulline concentration does not just reflect reduced synthesis by the small intestinal enterocytes [13], but may also be simultaneously increased by renal dysfunction [39], because most citrulline degradation occurs in the proximal tubules of the kidney. Hence, the degree of reduction in citrulline concentration may be curtailed in the presence of renal dysfunction, which in turn could contribute to a lack of a relationship between citrulline concentration and small intestinal glucose absorption. The mean creatinine concentration in the patients in this study was in the normal range, so this may not have been an important factor in most of the patients studied; nevertheless, it is a consideration, as four patients had abnormal renal function as defined by a creatinine level >100 μmol/L. Furthermore, the importance of renal failure as an influence on citrulline concentration in critical illness has been questioned [40].

Although it is frequently stated that citrulline concentration can be used to assess enterocyte function, few studies have quantified the relationship between citrulline and intestinal absorption, and those that have been performed have produced conflicting results. Our results are at odds with those reported by Papadia *et al*. [11]. They examined the relationship between citrulline concentration and xylose absorption using urine sampling in 55 patients with Crohn’s disease and short bowel syndrome. They found a strong correlation between bowel length and citrulline concentration and also between xylose absorption and citrulline concentration. There are a number of possible explanations for these conflicting results. First, it is possible, or indeed likely, that citrulline concentration reflects absorptive function in short bowel syndrome, but not in critical illness. Patients with short bowel syndrome will inevitably have impaired nutrient absorption directly related to the remaining length of short bowel, which will also reflect intestinal synthetic function. Patients who are critically ill may have reduced small intestinal synthetic function, but, as suggested above, their impaired absorption may have mechanisms other than reduced enterocyte mass. Another factor that may account for the differing results is the use of different techniques for the measurement of nutrient absorption. Xylose is a pentose sugar, whereas glucose is a hexose. The absorptive mechanism differs between the two agents, and there is redundancy in the absorption of glucose, making it much less likely to be affected by small intestinal pathology. Nevertheless, glucose absorption has repeatedly been shown to be impaired in patients who are critically ill [3,4], and the analysis of plasma concentration of 3-OMG is likely to be more clinically relevant, as glucose is a source of calories in enteral nutrition formulae. It is conceivable that if another probe is used, a relationship between citrulline and nutrient absorption may be demonstrated in patients who are critically ill. Citrulline concentration has been shown to have some correlation with fat (*r* = 0.53) and nitrogen (*r* = 0.47) absorption in short bowel syndrome [7]. Our results are consistent with those of a study of 24 patients with short bowel syndrome in whom macronutrient absorption, measured using acid hydrolysis and bomb calorimetry, did not correlate with citrulline concentration [15]. It appears that, although citrulline concentration may reflect enterocyte mass, it may not reflect enterocyte function in critical illness. Hence, unfortunately, citrulline cannot be used as an indicator of glucose absorption in critical illness.

A limitation of our study is that, although the sample size in the current study was sufficient to be able to demonstrate significant differences between critically ill patients and healthy subjects with regard to citrulline concentration and glucose absorption, it may have been underpowered to detect a relationship between citrulline concentration and glucose absorption. Although the diet of participants was not controlled prior to recruitment, the fasting period before data collection should have been sufficient to prevent any residual dietary effects on the outcomes, particularly because the only important source of citrulline is watermelon. A previous study showed that even administration of a high ‘dose’ of watermelon did not increase plasma citrulline concentration [41]. It is also possible that using a test sugar other than 3-OMG or another macronutrient and measurement of citrulline concentration on more than one occasion may have generated different results.

## Conclusions

This study confirms that fasting plasma citrulline concentration and glucose absorption are reduced in the critically ill when compared to healthy subjects. However, fasting plasma citrulline concentration does not appear to be a marker of subsequent glucose malabsorption. Accordingly, plasma citrulline concentration does not appear to be a robust measure of this aspect of small intestinal function in patients who are critically ill, but this may warrant further investigation with other macronutrients.

## Key messages

- Fasting plasma citrulline concentrations are reduced in critically ill patients compared to healthy subjects.
- Glucose absorption is reduced in critically ill patients compared to healthy subjects.
- Fasting plasma citrulline concentration does not appear to be a marker of glucose malabsorption.
- Although it has been suggested that citrulline is a biomarker of intestinal function, it does not appear to be a biomarker of glucose absorptive function.

## Abbreviations

3-OMG: 3-*O*-methylglucose
APACHE: Acute Physiology and Chronic Health Evaluation
ARDS: Acute respiratory distress syndrome
AUC: Area under the curve
BMI: Body mass index
ICU: Intensive care unit

## References

[1] Simpson F, Doig GS (2005). Parenteral vs. enteral nutrition in the critically ill patient: a meta-analysis of trials using the intention to treat principle. Intensive Care Med.

[2] Heyland DK, Dhaliwal R, Dover JW, Gramlich L, Dodek P (2003). Canadian clinical practice guidelines for nutrition support in mechanically ventilated, critically ill adult patients. JPEN J Parenter Enteral Nutr.

[3] Chapman MJ, Fraser RJ, Matthews G, Russo A, Bellon M, Besanko LK (2009). Glucose absorption and gastric emptying in critical illness. Crit Care.

[4] Deane AM, Summers MJ, Zaknic AV, Chapman MJ, Di Bartolomeo AE, Bellon M (2011). Glucose absorption and small intestinal transit in critical illness. Crit Care Med.

[5] Fordtran JS, Clodi PH, Soergel KH, Ingelfinger FJ (1962). Sugar absorption tests, with special reference to 3-*O*-methyl-d-glucose and d-xylose. Ann Intern Med.

[6] Levin RJ (1994). Digestion and absorption of carbohydrates—from molecules and membranes to humans. Am J Clin Nutr.

[7] Crenn P, Coudray-Lucas C, Thuillier F, Cynober L, Messing B (2000). Postabsorptive plasma citrulline concentration is a marker of absorptive enterocyte mass and intestinal failure in humans. Gastroenterology.

[8] Crenn P, Vahedi K, Lavergne-Slove A, Cynober L, Matuchansky C, Messing B (2003). Plasma citrulline: a marker of enterocyte mass in villous atrophy-associated small bowel disease. Gastroenterology.

[9] Curis E, Crenn P, Cynober L (2007). Citrulline and the gut. Curr Opin Clin Nutr Metab Care.

[10] Kaore SN, Amane HS, Kaore NM (2013). Citrulline: pharmacological perspectives and its role as an emerging biomarker in future. Fundam Clin Pharmacol.

[11] Papadia C, Sherwood RA, Kalantzis C, Wallis K, Volta U, Fiorini E (2007). Plasma citrulline concentration: a reliable marker of small bowel absorptive capacity independent of intestinal inflammation. Am J Gastroenterol.

[12] Pappas PA, Saudubray JM, Tzakis AG, Rabier D, Carreno MR, Gomez-Marin O (2002). Serum citrulline as a marker of acute cellular rejection for intestinal transplantation. Transplant Proc.

[13] Piton G, Manzon C, Cypriani B, Carbonnel F, Capellier G (2011). Acute intestinal failure in critically ill patients: is plasma citrulline the right marker?. Intensive Care Med.

[14] Piton G, Manzon C, Monnet E, Cypriani B, Barbot O, Navellou JC (2010). Plasma citrulline kinetics and prognostic value in critically ill patients. Intensive Care Med.

[15] Luo M, Fernández-Estívariz C, Manatunga AK, Bazargan N, Gu LH, Jones DP (2007). Are plasma citrulline and glutamine biomarkers of intestinal absorptive function in patients with short bowel syndrome?. JPEN J Parenter Enteral Nutr.

[16] Ware LB, Magarik JA, Wickersham N, Cunningham G, Rice TW, Christman BW (2013). Low plasma citrulline levels are associated with acute respiratory distress syndrome in patients with severe sepsis. Crit Care.

[17] Piton G, Belon F, Cypriani B, Regnard J, Puyraveau M, Manzon C (2013). Enterocyte damage in critically ill patients is associated with shock condition and 28-day mortality. Crit Care Med.

[18] Fone DR, Akkermans LM, Dent J, Horowitz M, van der Schee EJ (1990). Evaluation of patterns of human antral and pyloric motility with an antral wall motion detector. Am J Physiol.

[19] Cook CG, Andrews JM, Jones KL, Wittert GA, Chapman IM, Morley JE (1997). Effects of small intestinal nutrient infusion on appetite and pyloric motility are modified by age. Am J Physiol.

[20] Hutson WR, Roehrkasse RL, Wald A (1989). Influence of gender and menopause on gastric emptying and motility. Gastroenterology.

[21] Johansson C, Ekelund K (1976). Relation between body weight and the gastric and intestinal handling of an oral caloric load. Gut.

[22] Deane AM, Fraser RJ, Young RJ, Foreman B, O’Conner SN, Chapman MJ (2009). Evaluation of a bedside technique for postpyloric placement of feeding catheters. Crit Care Resusc.

[23] Heddle R, Dent J, Toouli J, Read NW (1988). Topography and measurement of pyloric pressure waves and tone in humans. Am J Physiol.

[24] Keshishian H, Addona T, Burgess M, Kuhn E, Carr SA (2007). Quantitative, multiplexed assays for low abundance proteins in plasma by targeted mass spectrometry and stable isotope dilution. Moll Cell Proteomics.

[25] Matthews JN, Altman DG, Campbell MJ, Royston P (1990). Analysis of serial measurements in medical research. BMJ.

[26] Peters JH, Beishuizen A, Keur MB, Dobrowolski L, Wierdsma NJ, van Bodegraven AA (2011). Assessment of small bowel function in critical illness: potential role of citrulline metabolism. J Intensive Care Med.

[27] Iresjö BM, Körner U, Larsson B, Henriksson BA, Lundholm K (2006). Appearance of individual amino acid concentrations in arterial blood during steady-state infusions of different amino acid formulations to ICU patients in support of whole-body protein metabolism. JPEN J Parenter Enteral Nutr.

[28] Déchelotte P, Hasselmann M, Cynober L, Allaouchiche B, Coëffier M, Hecketsweiler B (2006). l-alanyl-l-glutamine dipeptide-supplemented total parenteral nutrition reduces infectious complications and glucose intolerance in critically ill patients: the French controlled, randomized, double-blind, multicenter study. Crit Care Med.

[29] Jeevanandam M, Ramias L, Schiller WR (1991). Altered plasma free amino acid levels in obese traumatized man. Metabolism.

[30] Jeevanandam M, Young DH, Ramias L, Schiller WR (1989). Aminoaciduria of severe trauma. Am J Clin Nutr.

[31] Jensen GL, Miller RH, Talabiska DG, Fish J, Gianferante L (1996). A double-blind, prospective, randomized study of glutamine-enriched compared with standard peptide-based feeding in critically ill patients. Am J Clin Nutr.

[32] Sandstrom P, Trulsson L, Gasslander T, Sundgvist T, von Dobein U, Svanvik J (2008). Serum amino acid profile in patients with acute pancreatitis. Amino Acids.

[33] Yu YM, Ryan CM, Burke JF, Tompkins RG, Young VR (1995). Relations among arginine, citrulline, ornithine, and leucine kinetics in adult burn patients. Am J Clin Nutr.

[34] Long CL, Borghesi L, Stahl R, Clark JA, Geiger JW, DiRienzo DB (1996). Impact of enteral feeding of a glutamine-supplemented formula on the hypoaminoacidemic response in trauma patients. J Trauma.

[35] Noordally SO, Sohawon S, Semlali H, Michely D, Devriendt J, Gottignies P (2012). Is there a correlation between circulating levels of citrulline and intestinal dysfunction in the critically ill?. Nutr Clin Pract.

[36] Ochoa JB, Udekwu AO, Biliar TR, Curran RD, Cerra FB, Simmons RL (1991). Nitrogen oxide levels in patients after trauma and during sepsis. Ann Surg.

[37] Grimaldi D, Guivarch E, Neveux N, Fichet J, Pene F, Marx JS (2013). Markers of intestinal injury are associated with endotoxemia in successfully resuscitated patients. Resuscitation.

[38] Deane AM, Rayner CK, Keeshan A, Cvijanovic N, Marino Z, Nguyen NQ (2014). The effects of critical illness on intestinal glucose sensing, transporters, and absorption. Crit Care Med.

[39] Levillain O, Parvy P, Hassler C (1997). Amino acid handling in uremic rats: citrulline, a reliable marker of renal insufficiency and proximal tubular dysfunction. Metabolism.

[40] Cynober L (2013). Citrulline: just a biomarker or a conditionally essential amino acid and a pharmaconutrient in critically ill patients?. Crit Care.

[41] Collins JK, Wu G, Perkins-Veazie P, Spears K, Claypool PL, Baker RA (2007). Watermelon consumption increases plasma arginine concentrations in adults. Nutrition.

